# Adenovirus Type 7 Peptide Diversity during Outbreak, Korea, 1995–2000

**DOI:** 10.3201/eid1105.041211

**Published:** 2005-05

**Authors:** Eun Hwa Choi, Hee Sup Kim, Byung Wook Eun, Beyong Il Kim, Jung Yeon Choi, Hoan Jong Lee, Toshiki Inada

**Affiliations:** * Seoul National University College of Medicine, Seoul, Korea;; † Seoul National University Bundang Hospital, Kyungkido, Korea;; ‡ National Cancer Institute, Bethesda, Maryland, USA;; § Seoul National University Children's Hospital, Seoul, Korea;; ¶ National Institute of Infectious Diseases, Tokyo, Japan

**Keywords:** adenovirus type 7, fiber, E4 orf 6/7 peptides

## Abstract

To understand the molecular basis of observed regional shifts in the genome types of adenovirus type 7 (Ad7) isolated in Korea during nationwide outbreaks from 1995 to 2000, the genetic variabilities of Ad7d and Ad7l were studied by sequence analysis of hexon, fiber, E3, and E4 open reading frame (ORF) 6/7 peptides. One amino acid change in the receptor-binding domain of fiber and 6 amino acid variations in E4 ORF 6/7 were identified between 2 genome types, while no variations were found in hexon and E3. Phylogenetic trees based on hexon, fiber, and E4 suggested that the Ad7 epidemic was probably caused by the introduction of the Japanese Ad7d strains. Our data also provide evidence that the rapid divergence of Ad7d to a novel genome type Ad7l could have been due to viral strategies involving multiple sequence changes in E4. This result suggests fiber and E4 ORF 6/7 peptides participate in the evolution of Ad7.

Approximately 5% of upper respiratory tract infections and 8% of childhood pneumonia cases are attributed to adenoviral infection (1–4). Infection is generally restricted to the upper respiratory tract, but infection can sometimes develop in the lower tract or at other distal sites, such as kidney, heart, gastrointestinal tract, and eye. In particular, adenovirus serotype 7 (Ad7) has been associated with the most severe, often fatal, disease in children (4–7).

A nationwide outbreak of severe pneumonia caused by Ad7 occurred in Korea from 1995 to 2000 (7). Like other serotypes, diverse genome types within Ad7 have been identified by restriction enzyme analysis of the viral DNA (8). Two genome types, Ad7d and Ad7l, were recognized during this epidemic. Genome type Ad7l was described as a novel genome type and was found to be closely related to Ad7d based on unique *Bam*HI restriction patterns (9). Compared to their presence in strains of Ad7d, 2 restriction fragments (8,400 and 2,650 bp) are lost in strains of Ad7l, which contain a new fragment equal in length to the sum of those 2 fragments. The observed change in restriction pattern is the result of a mutation of the *Bam*HI restriction site at ≈0.93 map units of the genome. This site falls at the 3´ end of the open reading frame (ORF) 6/7 peptides of the early region 4 (E4) (unpub. data).

Different Ad7 genome types have predominated in different areas during the last 3 decades (5–8,10,11). Particularly interesting were the distinctive patterns of circulating genome types during the Ad7 epidemic in Korea and in 3 neighboring countries, China, Taiwan, and Japan. Ad7d was identified as early as 1980 in Beijing and was subsequently replaced by Ad7b, which then became the predominant genome type in China through the 1990s (5). In southern Taiwan, a shift from Ad7a to Ad7b was reported from 1983 to 2000 (12). Ad7d was also identified in Japan from 1987 to 1992, and Ad7d2 was the major genome type isolated during a large Japanese outbreak from 1995 to 1998 (13). In Korea, genome type Ad7d was predominantly observed at the beginning of the 1995–1997 epidemic, but it was rapidly replaced by a novel genome type Ad7l from 1998 to 2000 (9).

Both epidemiologic and molecular evidence strongly suggest that unique patterns of genome type shifts are restricted to geographic areas. However, why some Ad7 genome types (e.g., Ad7d, Ad7d2, and Ad7h) are able to spread globally while others (e.g., Ad7i) are limited to part of the world (11) is not well understood. Previously, sequence variations among the different genome types of Ad7 strains have been observed at 2 variable regions of the hexon gene and in a 14.9-kDa protein encoded by an ORF in the E3 region (6,14).

This study was undertaken to determine genetic differences and to understand the molecular basis of regional shifts observed in the genome types of Ad7 isolated in Korea. Genetic variations between the Ad7d and Ad7l genome types have been studied by analyzing the sequences of hexon, E3, fiber, and E4 ORF 6/7 peptides. Fiber and E4 ORF 6/7 peptides were chosen for study because of close proximity to a mutation site at 0.93 map units, and hexon and E3 peptides were chosen because of their reported genetic heterogeneities.

## Methods

### Virus Analysis

Twelve of the 98 Ad7 isolates obtained from Korean children with pneumonia from 1995 to 2000 were subjected to genetic analysis. Seven strains of genome type Ad7d and 5 strains of Ad7l were selected from various places and different times to represent epidemiologically unrelated strains (Table 1). Full-length adenoviral DNA was purified from infected Hep-2 cell lysates by using a modified Hirt procedure, as previously described (15). Genome types were assigned by restriction fragment analysis with 12 enzymes as in the previous study (9).

**Table 1:** Twelve selected strains of adenovirus type 7 isolated from children with pneumonia during a nationwide outbreak, Korea, 1995–2000

Strain name	Genome type	Isolation	
Place	Date
95081	7d	Seoul	Oct 1995
96241	7d	Kyungki Province	Jun 1996
96260	7l	Pusan	Jul 1996
96285	7d	Kyungsang Province	Jul 1996
96373	7d	Seoul	Nov 1996
97010	7d	Kyungsang Province	Jan 1997
97215	7l	Chungchung Province	Jul 1997
98234	7l	Kyungki Province	Jun 1998
98330	7d	Chulla Province	Jul 1998
98422	7l	Seoul	Aug 1998
98649	7d	Seoul	Nov 1998
99095	7l	Seoul	Jan 1999

### Sequence Analysis

**Figure 1:**
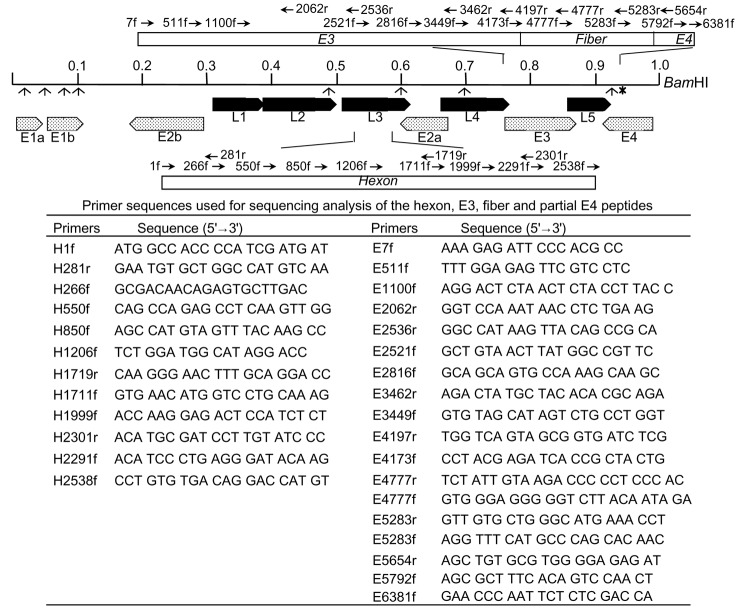
Schematic representation of the restriction mapping sites of adenovirus type 7d (Ad7d) and Ad7l by *Bam*HI, the primer sequences, and their location for the sequencing analysis of hexon, E3, fiber, and E4 open reading frame (ORF) 6/7 peptides. Each restriction site by *Bam*HI is indicated as (↑). The restriction site at 0.93 map units shown by (↑*) is lost in strains with genome type Ad7l and present in Ad7d. Figure of each primer represents H, hexon; E, E3, fiber; and E4 ORF 6/7 peptides, the position of the first nucleotide at the 5´ end; f, forward; and r, reverse. The numbering of nucleotides is based on that of strain 383, which was isolated in Japan in 1992 (AF053086 for hexon and AF104383 for E3, fiber, and E4).

The entire sequences of hexon, E3, fiber, and E4 ORF 6/7 peptides were determined. Adenoviral DNA (1–2 μg) was used as a template for sequencing. Sequencing primers were designed from GenBank reference sequences (GenBank accession no. AF053086 for the hexon gene; AF104383 for the E3, fiber, and E4 ORF 6/7 peptides). Nucleotide sequences were confirmed by duplicate reactions by using the primers shown in Figure 1.

Sequence analysis was performed by using a dideoxy chain termination reaction with BigDye Terminator (Applied Biosystems, Inc, Foster City, CA, USA), and run on an ABI 377 or ABI 3700 automated sequencer (Applied Biosystems, Inc). Data were analyzed with Sequencing Analysis v. 3.3 (Applied Biosystems, Inc). Chromatograms were imported into Sequencher 4.1.1 (Gene Codes Co, Ann Arbor, MI, USA) for assembly into contigs and for variation identifications. Both nucleotide and predicted amino acid sequences were aligned by using the ClustalW 1.4 method (http://www.ebi.ac.uk/clustalw/).

### Nucleotide Sequence Accession Numbers

The sequences of hexon, E3, fiber, and E4 ORF 6/7 peptides of Korean strains were compared to those previously reported for Ad7 strains in GenBank. The sequences obtained during this study were registered with the GenBank database under the following accession numbers: the hexon gene, AY769945 for Ad7d and AY769946 for Ad7l; fiber and E4 ORF 6/7 peptides, AY921615 for Ad7p (Gomen strain), AY921616 for Ad7a (strain S-1058), AY921618 for Ad7d (strain 383), AY921620 for Ad7d (strain Bal), AY921621 for Ad7d (strain 95081), AY921622 for Ad7d (strain 98330), and AY921617 for Ad7l (strain 99095).

### Phylogenetic Analysis

To understand the evolutionary process underlying the regional shift in the genome type of Ad7 in Korea, phylogenetic relationships were analyzed by using 1,428 bp making up nucleotides (nt) 297–1725 of the hexon gene. Another phylogenetic tree was generated based on 2,150 bp of fiber and E4 ORF 6/7 peptides. The phylogenetic trees constructed included sequences of hexon in 17 strains of Ad7 with diverse genome types and those of the fiber with E4 for only 7 strains, as available sequences were limited. Analysis was conducted by using MEGA version 2.0 (16). Kimura 2-parameters were used for the distance method, by using the neighbor-joining algorithm. Five hundred additional bootstrap analyses were performed on each phylogenetic tree.

## Results

The complete sequence of the hexon (2,805 bp, 934 amino acids [aa]), E3 peptides (4,387 bp), the fiber gene (1,175 bp, 325 aa), and E4 ORF 6/7 peptides was successfully determined for 12 strains of Ad7.

### Hexon Gene

No variations were found in the complete sequence of the hexon gene between the 7 strains of Ad7d and 5 of Ad7l isolated in Korea. Moreover, their sequences were identical to the previously published sequences of the Japanese Ad7d (AF053086, strain 383) and Ad7d2 (AF053087, strain Bal) strains. The Korean Ad7 isolates belonged to genome type cluster 2 based on the 2 hypervariable regions (14). Substitution of Leu with Gln at aa 440 of loop 2, which dramatically affects the hydropathic character of this region, was also observed in 12 Korean Ad7 strains. In terms of the 473 aa from position 100 to 572 on the hexon gene, 8 mutation sites were observed on comparing Korean Ad7 strains obtained from 1995 to 1999 and Chinese Ad7d isolates from 1981 to 1984 (Table 2). Ad7 strains with different genome types recently isolated from Korea and Japan shared identical sequences, whereas 4 Chinese Ad7d strains showed 8 aa changes within 1 genome type.

**Table 2:** Comparison of 473 amino acid sequences from position 100 to 572 of the hexon gene in adenovirus type 7 strains

Genome type	Strain name	Place of origin (y)	Accession no.	Amino acid position*								
100	166 (169)	197 (200)	220 (223)	265 (268)	284 (287)	348 (351)	508 (511)	519 (522)
7p	Gomen	USA (1954)	Z48571†	Phe	Ile	Thr	Gly	Ala	Ser	Gln	Leu	His
7a	S-1058	USA (1955)	AF053085	Phe	Ile	Thr	Gly	Ala	Ser	Gln	Leu	His
7d	BC3655	China (1981)	U77392	Cys	Ile	Ile	Gly	Thr	Arg	Arg	Phe	His
7d	BC4492	China (1984)	U75953	Tyr	Ile	Ile	Gly	Ala	Ser	Gln	Leu	His
7d	BC4609	China (1984)	U77393	Tyr	Ile	Ile	Gly	Ala	Ser	Gln	Leu	Tyr
7d	BC8488	China (1984)	U77394	Tyr	Val	Ile	Arg	Ala	Ser	Gln	Leu	His
7d	383	Japan (1992)	AF053086	Phe	Ile	Ile	Gly	Ala	Ser	Gln	Leu	His
7d2	Bal	Japan (1995)	AF053087	Phe	Ile	Ile	Gly	Ala	Ser	Gln	Leu	His
7d	95081	Korea (1995)	AY769945	Phe	Ile	Ile	Gly	Ala	Ser	Gln	Leu	His
7l	99095	Korea (1999)	AY769946	Phe	Ile	Ile	Gly	Ala	Ser	Gln	Leu	His

*Numbers in parentheses indicate the amino acid positions of Ad7p, Gomen strain. The remaining 25 amino acid (aa) changes specific to Gomen strain and 1 aa change specific to S-1058 are not provided in this table. †Complete genome of the Gomen strain is available at GenBank (accession no. AY594255).

### E3 Region

The sequencing of 4,387 nt of the E3 region demonstrated no nucleotide substitutions within 12 Korean Ad7 strains. Thus, no sequence differences associated with amino acid changes were found in the E3 region of Ad7d and Ad7l. In particular, the amino acid sequence of Ad7d and Ad7l was conserved at codon 89 in the ORF encoding a 14.9-kDa segment of the E3 region, where Gly was substituted with Ser in the Japanese strains of Ad7d2.

### Fiber Gene

We analyzed 975 bp of the fiber gene. Two variations were detected in the amino acid sequences of fiber of Korean Ad7 versus previously reported Japanese strains. These were located in the receptor-binding domain, the so-called knob region. Compared to the Japanese isolates of Ad7d and Ad7d2, all Korean Ad7 strains showed substitution of Ala to Val at codon 112. Within Korean Ad7, a change of Arg to Lys at codon 280 was observed in 4 strains of Ad7d and all Ad7l strains (Table 3). However, this variation was not correlated with genome type. Analysis of the hydrophobicity of this mutation site showed minimal influence on hydropathic characters.

**Table 3:** Comparison of amino acid sequences of fiber and E4 open reading frame (ORF) 6/7 peptides among adenovirus type 7 strains

Genome type	Strain name	Accession no.	Amino acid position*									
			Fiber			33.2 kDa of E4						9.4 kDa of E4
			104	112	280	12	94	110	128	191	258	38
7p	Gomen	AY921615†	Glu	Ala	Arg	Arg	Arg	Asn	Arg	Ile	Val	Phe
7a	S-1058	AY921616	Glu	Ala	Arg	Arg	Arg	Asn	Gln	Ile	Val	Phe
7d	383	AY921618	Gly	Ala	Arg	Arg	Lys	His	Arg	Ile	Val	Phe
7d2	Bal	AY921620	Gly	Ala	Arg	Arg	Lys	His	Arg	Ile	Val	Phe
7d	95081	AY921621	Gly	Val	Lys	Arg	Lys	His	Arg	Ile	Val	Phe
7d	98330	AY921622	Gly	Val	Arg	Arg	Lys	His	Arg	Ile	Val	Phe
7l	99095	AY921617	Gly	Val	Lys	His	Lys	Asn	Gln	Leu	Ala	Ser

*The remaining 5 amino acid changes, which are specific to Gomen strain only, are not provided in this table. †Complete genome of the Gomen strain is available at GenBank (accession no. AY594255).

### ORF 6/7 Peptides of E4

Nucleotide sequences were determined for the 33.2-kDa and 9.4-kDa peptides of the E4 ORF 6/7. On comparing 7 strains of Ad7d, 5 strains of Ad7l showed nucleotide substitutions at 18 sites, including a mutation site at 0.93 map units. Of these 18 sites, 6 resulted in amino acid changes as shown in Table 3. Nucleotide identity between Ad7d and Ad7l was 98.4%. Comparisons of the hydrophobicity plots of these 6 aa variations demonstrated small hydropathic changes.

To exclude possibility that only the 12 isolates examined in the present study differed with respect to the residues of E4 ORF 6/7 peptides, an additional 28 isolates (15 of Ad7d and 13 of Ad7l) were also sequenced for this region. The sequencing results consistently showed identical nucleotide changes at the 18 sites in the Ad7d and Ad7l genome types.

### Phylogenetic Relationships

**Figure 2:**
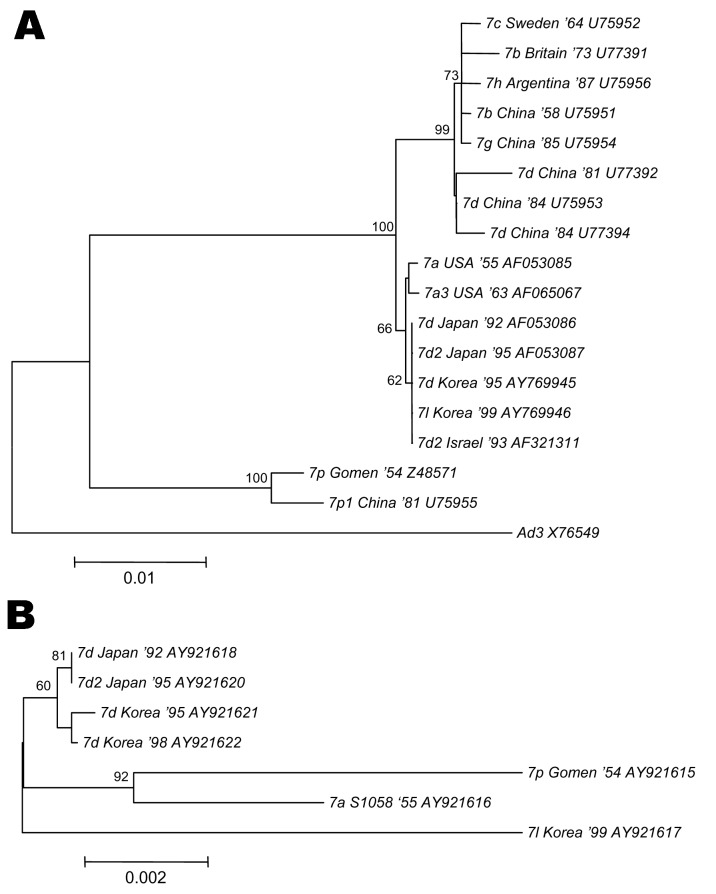
Phylogenetic analyses of both hexon and fiber with E4 open reading frame (ORF) 6/7 peptides in adenovirus type 7 (Ad7). Phylogenetic trees were constructed by using the neighbor-joining algorithm. Branch lengths are proportional to the number of nucleotide substitutes, and bootstrap probabilities ≥60 are shown at each adjacent node. A) Phylogenetic tree based on 1,428 bp making up nucleotides 297–1725 of the hexon gene of 17 strains of Ad7 with diverse genome types. The sequence of Ad3 (X76549) was defined as an outgroup. B) Phylogenetic tree based on 2,150 bp, including the complete sequence of fiber and E4 ORF 6/7 peptides.

To understand the distinct pattern of evolutionary relationship between Ad7 strains, phylogenetic relationships were inferred based on the sequences of hexon and fiber with E4 ORF 6/7 peptides (Figure 2). A phylogenetic tree based on the hexon sequence showed 2 distinct clusters, 1 of which subdivided into 2 lineages. Ad7 isolates from both Korea and Japan were clustered into the same lineage, regardless of genome type, since the sequences were identical. This cluster was distinct from other lineages, including the Chinese Ad7d and other genome types such as Ad7p, Ad7b, Ad7c, Ad7g, and Ad7h. Phylogenetic trees based on the fiber and E4 ORF 6/7 peptides demonstrated that genome type Ad7l showed remarkable changes within this region compared to the related genome types, Ad7d and Ad7d2.

## Discussion

We identified previously unrecognized variations in the fiber gene and E4 ORF 6/7 peptides among the various genome types of Ad7. In particular, multiple amino acid changes at E4 ORF 6/7 peptides showed genome type–specific differences between Ad7d and a novel genome type Ad7l. However, no genetic divergence was identified in the hexon gene or in E3 peptides among the 12 Korean strains of Ad7. This result indicates that the nucleotide structures of the fiber gene and of E4 ORF 6/7 peptides might have contributed to the genetic heterogeneity during the Ad7 epidemics in Korea, whereas those of the hexon gene and E3 were highly conserved.

So far, the fiber gene and E4 have rarely been addressed with regard to the evolution and molecular epidemiology of Ad7 strains (17). Restriction analysis of genomic DNA has been the most popular method of describing the molecular epidemiology of the adenoviruses (18). However, little is known about sequence variations with regard to the genome type within an epidemic or between the epidemics. Previous observations indicate that sequence variations of the hexon gene and of the E3 ORF encoding 14.9-kDa protein may contribute to genetic heterogeneity and the evolutionary process of Ad7 strains (6,14,19,20).

Alignment of the amino acid sequences of the hexon gene demonstrated 2 genetic clusters, GTC1 (Ad7p and Ad7p1) and GTC2 (Ad7a, Ad7b, Ad7c, Ad7d, Ad7d2, Ad7g, and Ad7h), based on variations in the hypervariable regions (14). Variations between 2 clusters have been observed in variable region 1 because of 3-aa length differences and in the variable region 2 because of a substitution from 440 Leu to Gln, the latter of which dramatically affected the hydropathic character of this region. Moreover, mutations of hexon of Ad could play an important role in new outbreaks of adenoviral infection (14). Compared to the Chinese Ad7d strains of the early 1980s, 1 aa substitution from Cys (or Tyr) to Phe at position 100 was consistently observed in all Korean isolates among 8 mutation sites. However, the Ad7d and Ad7l Korean strains shared the same hexon gene sequence as Japanese Ad7d and Ad7d2 in 1992 and 1995. Phylogenetic tree analysis based on the hexon sequences suggested that the Ad7 isolates from Korea and Japan cluster into the same lineage and that this cluster is distinct from those of Chinese Ad7d and other strains. Therefore, the Korean Ad7d epidemic that began in 1995 may have been caused by the introduction of isolates that were prevalent in Japan from 1987 to 1992.

Fiber is a major constituent of adenovirus outer capsid (21). Fiber protein consists of a trimeric projection terminated by a knob (head) (21,22), and it plays a crucial role in adenoviral infection by allowing, possibly by direct interaction, the virus to attach to specific receptors on the host cell surface (23). In addition, variability in this region is expected to account for the observed serologic difference between serotype 3 and 7 fibers (24). Two variation sites observed in Ad7 strains are located at the knob region of the fiber gene near its carboxy-terminal end. All Korean Ad7 strains showed a substitution of Ala to Val at codon 112 with or without a change of Arg to Lys at codon 280, unlike the Japanese strains. Therefore, the Korean Ad7d strains are thought to be closely related to the Japanese strains with changes in the receptor domain of the fiber gene. Changes in the receptor-binding domain could influence the interaction between fiber and the host cell and possibly influence genetic heterogeneity in different geographic locations.

The E4 region of human adenoviruses encodes a set of proteins that can regulate early gene expression for viral RNA export and stabilization (21,25). The functions of E4 ORF 6/7 peptides of Ad7 are unknown, but the corresponding protein of adenovirus type 5 has the ability to induce the binding of cellular transcription factor E2F to the viral E2a promoter region (26). E2F induced by ORF 6/7 peptides might facilitate adenoviral infection under more stringent environments, such as in the absence of the E1A gene products (27,28). Furthermore, the 33.2-kDa ORF 6 protein is known to promote cell cycle–independent adenovirus growth (29).

Nucleotide changes specific to a novel genome type Ad7l were observed at 18 sites in E4 ORF 6/7 peptides. These substitutions have not been previously reported in published E4 sequences of Ad7 isolates from other parts of the world. Although available sequences are limited, a phylogenetic tree based on the sequences of fiber and E4 ORF 6/7 peptides showed evidence that genome type Ad7l has experienced substantially more changes than Ad7d strains. This finding strongly suggests that the E4 region can be important to molecular diversity and evolution of the Ad7 strains. Interplay between the host cell and the E4 of Ad7 may contribute to the observed distinctive features of genome types.

A total of 270 adenoviruses were identified from childhood pneumonia patients in our children's hospital during 10 years, 1990–2000. Of these 270 strains, Ad7d was not isolated until 1995, when explosive outbreaks occurred. Although we do not know the level of herd immunity to Ad7 at the beginning of epidemics, Ad7 had rarely circulated in Korea before the outbreak described in this study. Therefore, we assume that low immunity to Ad7 was critical to causing a large outbreak of Ad7. However, Noda et al. also reported that the mutation in the E3 of Ad7d2 strains could contribute to rapid spread during nationwide outbreaks in Japan (6).

As noted earlier, the nationwide outbreak of Ad7d in Korea in 1995 may have started with the introduction of the Japanese Ad7d strains, likely several years before 1995. To achieve a large outbreak, new Ad7d strains presumably required time to acquire greater access to hosts or the increased virulence acquired by fiber amino acid changes. However, a strain of novel genome type Ad7l was first detected in 1996, a year after the start of the Ad7d nationwide epidemic, spread rapidly during subsequent years, and then became the predominant genome type from 1998 to 2000. As reflected by their capacity to displace Ad7d, Ad7l might be more efficient than Ad7d for the dissemination in nonimmune hosts when a certain level of immunity was maintained. Whether the variations influence viral capacity for virulence and transmission during the epidemics is not currently understood; however, data shown in this study suggest that Ad7l was rapidly spread by multiple amino acid changes at fiber and E4 ORF 6/7 peptides during Ad7 outbreaks in Korea. This process might have been a consequence of viral adaptive strategies that allowed the virus to spread throughout Korea before the population had developed immunity.

In conclusion, the results presented here emphasize that fiber and E4 ORF 6/7 peptides may have roles in the evolutionary process and pathogenesis of Ad7 in Korea. The emergence of new genome types after the disappearance of a previously predominant type may be the result of type-specific host immune response or type-specific virulence, perhaps mediated by amino acid variations in fiber or E4. However, the mechanisms underlying viral adaptive processes and interactions between virus and host have to be established by future study to allow us to more effectively counteract highly virulent genome types.
